# Preparation and Antiproliferative Activity Evaluation of Juglone-Loaded BSA Nanoparticles

**DOI:** 10.34172/apb.2022.087

**Published:** 2022-01-03

**Authors:** Ali Jahanban-Esfahlan, Soodabeh Davaran, Siavoush Dastmalchi

**Affiliations:** ^1^Biotechnology Research Center, Tabriz University of Medical Sciences, Tabriz, Iran.; ^2^School of Pharmacy, Tabriz University of Medical Sciences, Tabriz, Iran.; ^3^Drug Applied Research Center, Tabriz University of Medical Sciences, Tabriz, Iran.; ^4^Faculty of Pharmacy, Near East University, POBOX: 99138, Nicosia, North Cyprus, Mersin 10, Turkey.

**Keywords:** BSA, Cancer, Chemotherapy, Drug delivery, Juglone, Nanocarriers, Protein

## Abstract

***Purpose:*** Today, the discovery of novel and effective chemotherapeutic compounds is the main challenge in cancer therapy. In recent years, the anti-tumoral activity of natural naphthoquinone juglone (JUG), present in different parts of walnut trees, has received considerable interest. The purpose of the current study was to prepare and evaluate the *in vitro* antiproliferative activity of JUG-loaded bovine serum albumin nanoparticles (JUG-BSA NPs).

***Methods:*** BSA NPs and JUG-BSA NPs were prepared using the desolvation technique. The NPs were characterized for their particle size (PS), zeta potential (ZP), drug loading (DL) capacity and encapsulation efficiency (EE). The anti-proliferative activity of JUG-BSA NPs was evaluated on A431 and HT29 cancer cell lines using cellular uptake and MTT assays.

***Results:*** The PS and ZP values of JUG-BSA NPs were 85 ± 6.55 nm and −29.6 mV, respectively. The DL capacity and EE were 3.7% to 5% and 50.4% to 94.6%, respectively. The cytotoxicity of JUG-BSA NPs was significantly less on both cultured A431 and HT29 cells at the studied concentrations when compared to free JUG. However, the effect was not very substantial, particularly at high levels.

***Conclusion:*** In conclusion, BSA NPs can be used as a suitable and safe carrier for the delivery of JUG, a cytotoxic hydrophobic natural compound.

## Introduction

Cancer remains a leading cause of death worldwide. Despite recent advances in radiation and surgical treatments, chemotherapy remains an essential therapeutic approach to the treatment of patients with metastatic cancers. However, some problems associated with chemotherapy such as drug resistance, toxic side effects and the bioavailability of chemotherapeutic agents often restrict the drug concentration used to eliminate cancer cells. Specific targeted drug delivery could reduce the systemic toxicity of chemotherapeutic agents on healthy cells and increase the efficacy of anticancer drugs.^1,2^

**Figure 1:**
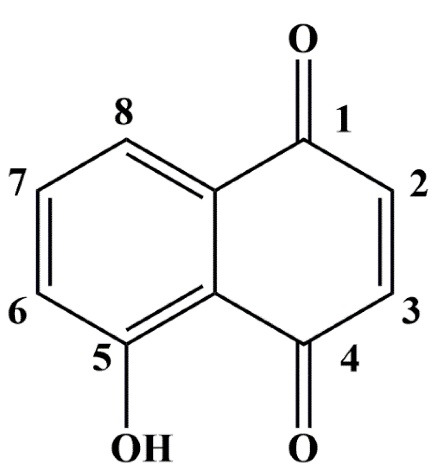
The chemical structure of JUG

Quinones are a broad category of widely distributed natural quinoid compounds, whose properties include anti-tumoral activity.^3,4^ JUG (5-hydroxy-1, 4-naphthoquinone) (Figure 1) is a natural naphthoquinone present in the leaf, root, kernel, skin, shell, husk, bark and wood of *Juglandaceae* family members such as Manchurian walnut (*Juglans mandshurica*), black walnut (*Juglans nigra*), common walnut (*Juglans regia*) and butternut (*Juglans cinerea*) trees.^5,6^ The bark, branches and the exocarp of the immature green fruit of these medicinal plants have been widely used for the treatment of gastric, liver, lung and other types of cancer in Chinese medicine and inhibits intestinal carcinogenesis induced by azoxymethane in rats and might be a promising chemo-preventive agent in human intestinal neoplasia.^7^ JUG is also a potent cytotoxic compound *in vitro* on various human tumor cell lines.^8-16^ Nevertheless, JUG has cytotoxic effects on normal healthy cells by different mechanisms.^17-20^ The side effects, as well as low water solubility, restrict the application of JUG as an antitumor agent rationalizing the studies to rectify these problems. It has been shown that JUG can interact with both human serum albumin (HSA) and bovine serum albumin (BSA) on the subdomain IA via the hydrogen bonds and hydrophobic forces.^21^ Thus, JUG can be loaded or entrapped in the albumin nanoparticles (JUG-BSA NPs) using a simple desolvation procedure without the preparation of drug-carrier conjugate. The use of albumin for the fabrication of NPs has been received increasing interest. Albumin is an acidic, very soluble protein, soluble in 40% ethanol, stable in the pH range of 4–9, and can be heated at 60°C for up to 10 hours without harmful effects.^22-24^ These properties, as well as its ready availability, preferential uptake in tumors and inflamed tissues, biodegradability, lack of toxicity and immunogenicity make it an ideal candidate for the targeted delivery of various hydrophobic or hydrophilic drugs.^25-28^ Abraxane as the first HSA-based NP with the mean particle size (PS) of 130 nm was approved by the Food and Drug Administration (FDA) in 2005 for the delivery of anticancer agent paclitaxel (PTX). It has been shown that the presence of HSA in the new formulation increases the solubility of the drug, decreases its side effects and enables the targeted delivery to tumor sites over the conventional PTX therapy.^29,30^

In the present investigation, BSA was used as a carrier for the preparation of BSA NPs and the possible delivery of JUG. A431, a human epidermoid carcinoma cancer cell line and HT29, a human colorectal adenocarcinoma cell line were considered for evaluating the antiproliferative activities of JUG-BSA NPs. Among different types of cancer, squamous cell carcinoma, also identified as epidermoid carcinomas, includes various types of cancers resulting from squamous cells. These cells form on the lining of the digestive and respiratory tracts, as well as the surface of the skin, and the covering of hollow organs in the body. Colorectal cancer is known as colon, bowel, or rectal cancer, in which oncogenic cells develop in the rectum (a part of the large intestine) or colon.^31^ Generally, in terms of incidence and mortality, colorectal cancer ranks third and second worldwide, respectively.^2^ To the best of the our knowledge, only one study on the formulation of JUG and evaluation of its anti-tumoral activity has been reported.^32^ Therefore, in the current study, we prepared and evaluated the *in vitro* anti-tumoral properties of JUG-BSA NPs.

## Materials and Methods

###  Chemicals

 JUG (purity 98.0%) was purchased from ACROS (New Jersey, USA). BSA was from Sigma-Aldrich (Steinheim, Germany). Sorbitol (cryo-protectant sugar) and N-(3-dimethyl aminopropyl)-N-ethyl carbodiimide hydrochloride (EDC), the crosslinking agent, were obtained from Merck (Schuchardt OHG, Hohenbrunn, Germany). MTT (3-(4,5-dimethylthiazol-2-yl)-2,5-diphenyltetrazolium bromide) powder was obtained from Sigma (St. Louis, USA). A431 and HT29 cell lines were obtained from the Pasture Institute of Iran in Tehran. RPMI-1640 modified medium was purchased from Gibco Invitrogen (Carlsbad, CA, USA). Water used for all experiments was of ultrapure prepared by a Milli-Q water purification system (Millipore, USA).

###  Preparation of BSA and JUG-BSA NPs 

BSA NPs were prepared by the desolvation technique as described, with minor modifications.^33^ Briefly, BSA (250 mg) was dissolved in water (4 mL). At a rate of 2 mL/min under constant stirring (1250 rpm), 8 mL ethanol was added to the solution at room temperature. The development of turbidity in the solution showed the formation of the NPs. To stabilize the formed particles, 0.5 mL freshly prepared 1% (w/v) aqueous solution of EDC was added and the stirring was continued for a further 3h to ensure complete crosslinking. JUG-BSA NPs were prepared by dissolving different amounts of JUG in the 8 mL ethanol and following the rest of the protocol as outlined above for BSA NPs. Three different formulations were prepared by keeping the concentration of albumin constant while varying the concentration of JUG (Table 1).

**Table 1:** The amounts of BSA and JUG used for the preparation of JUG-BSA NPs and also the calculated DL and EE for the prepared JUG -BSA NPs

**No.**	**BSA (mg)**	**JUG (mg)**	**DL (%)**	**EE (%)**
1	250	25	5.0 ± 0.2	50.4 ± 2.6
2	250	12.5	4.1 ± 0.5	82.9 ± 11.9
3	250	10	3.7 ± 0.1	94.6 ± 2.5

###  Purification of NPs

 The prepared BSA and JUG-BSA NPs were purified by three cycles of ultracentrifugation (Beckman Optima TLX Ultracentrifuge, USA) at 20 000 and 15 000 rpm, respectively for 10 minutes at 25°C followed by redispersion of the pellet in water to the original volume. The coacervates obtained after centrifugation were lyophilized using sorbitol (5%) as a cryoprotectant to obtain a fine powder of the nanoformulation.

###  Determination of encapsulation efficiency and drug-loading

The drug-loading (DL) capacity of JUG-BSA NPs was determined with an indirect method by collecting the supernatant of the purified NPs. The amount of unloaded free JUG present in the supernatant was determined by UV spectrophotometer (CECIL 8000 SERIES, USA) at 247 nm using a standard calibration curve plotted for JUG (R^2^ = 0.9986). The percentages of encapsulation efficiency (EE) and DL were calculated using the following equations:


EE00=Weight of the JUG loaded in NPsWeight of the JUG feeded×100

DL00=Weight of the JUG loaded in NPsWeight of NPs solid mass×100

###  Determination of free albumin concentration after desolvation

The amount of unincorporated BSA in JUG-BSA NPs was determined using the Bradford assay.^34^ Briefly, the prepared BSA and JUG-BSA NPs were separated from the supernatant by ultracentrifugation (Beckman Optima TLX Ultracentrifuge, USA) at 20 000 and 15 000 rpm, respectively for 10 minutes at 25°C. An aliquot of the supernatant (100 µL) was diluted with 5 mL Coomassie Brilliant Blue reagent (10 mg in 100 mL ethanol) and the samples were vortexed immediately. After 5 minutes, the absorbance was recorded using a UV spectrophotometer (CECIL 8000 SERIES, USA) at 595 nm. The protein content of the samples was determined by a calibration curve prepared using BSA standard solutions (R^2^ = 0.9992).

###  Particle size and zeta potential 

The PS of the prepared BSA and JUG-BSA NPs was measured by laser light scattering technique using a PS analyzer (Wing SALD 2101, Japan) as outlined elsewhere.^31^ Briefly, BSA and JUG-BSA NPs suspensions were diluted in water and PS analysis was performed under continuous stirring. The zeta potential (ZP) of both prepared BSA and JUG-BSA NPs were measured by a dynamic light scattering technique using Malvern Zetasizer Nano ZS (Malvern Instruments, UK).

###  Morphological analysis of NPs 

 Morphological features like sphericity and aggregation of the prepared BSA and JUG-BSA NPs were analyzed by scanning electron microscopy (SEM; Vega Tescan, Czech Republic). Briefly, lyophilized powder samples of NPs were coated with gold under vacuum and then analyzed.

###  Physical stability 

 JUG-BSA NPs samples were stored in PBS buffer pH 7.4 at room temperature. After two weeks, 200 μL NPs suspension was diluted to 12 mL using ultrapure water and the PS was determined using a PS analyzer (Wing SALD 2101, Japan). To determine the amount of JUG released from the NPs, 1 mL stored NPs suspension was centrifuged using ultracentrifuge (Beckman Optima TLX Ultracentrifuge, USA) and then the amounts of leaked JUG were determined in the supernatant using UV spectrophotometry (CECIL 8000 SERIES, USA) at 247 nm.

###  Cell culture 

A431 and HT29 cell lines were cultured in T-25 flasks at 37°C in a humidified incubator containing 5% CO_2_ using RPMI-1640 medium supplemented with 10% heat-inactivated fetal bovine serum and 1% penicillin-streptomycin. The cell culture medium was replaced every day.

###  Fluorescence microscopy 

The cellular internalization of the prepared JUG-BSA NPs was investigated by fluorescence microscopy.^35^ A431 and HT29 cells were cultured at a density of 300000 cells per well in 6-well plastic dishes for 24 h. The NPs were added to the cell culture medium at a concentration of 1 mg/mL. Additionally, the cells were also subjected to the suspension of BSA NPs and JUG solution diluted with a culture medium. After 4 hours of incubation at 37°C, the cells were washed three times with PBS and fixed with 10% glutaraldehyde in PBS. The cellular uptake of JUG-BSA NPs, BSA NPs and free JUG was observed by fluorescence microscopy (Olympus BX 50, Japan).

###  In vitro cytotoxicity studies 

The MTT assaywas employed to investigate the* in vitro* cytotoxicity of BSA NPs, JUG-BSA NPs and JUG on A431 and HT29 cells. Briefly, both A431 and HT29 cells seeded in 96-well plates at a density of 10 000 cells per well were incubated for 24 hours. For the free JUG, a stock solution was prepared in ethanol (1 mM), and appropriate concentrations were obtained by dilution from the stock solution in the culture medium. The suspensions of BSA and JUG-BSA NPs were diluted in culture medium at equivalent JUG concentrations ranging from 25–100 µM. The cell culture medium was then removed and replaced by fresh medium containing various amounts of BSA NPs, JUG-BSA NPs and JUG. The cells were incubated for 24, 48 and 72 hours at 37°C. After this, 20 μL MTT (5 mg/mL PBS) was added to each well. After 3 h, the medium containing MTT was discarded and 200 µL DMSO and 25 µL Sorenson buffer (0.1 M glycine, 0.1 M NaCl, pH 10.5) were added to each well to dissolve the formazan crystals and then the absorbance was recorded using BioTek ELx 800 plate reader (Biotek, CA, USA) at 570 nm. Finally, cell viability was obtained using the following formula:


$$\%\;of\;surviving\;cells=\frac{OD\;of\;well\;with\;treated\;cells}{OD\;of\;well\;with\;untreated\;cells}\times 100$$

The absorbance of control wells was obtained from the untreated cells (mean of three wells). All treated cells were also assayed at least in triplicates, and the results were expressed as the mean percentage of surviving cells.

###  Statistical analysis

All the experiments were performed in triplicate and the results were expressed as mean ± standard error (SE). SPSS software was used for data analysis and *P* < 0.05 was considered statistically significant.

## Results and Discussion

###  Preparation of NPs 

**Figure 2:**
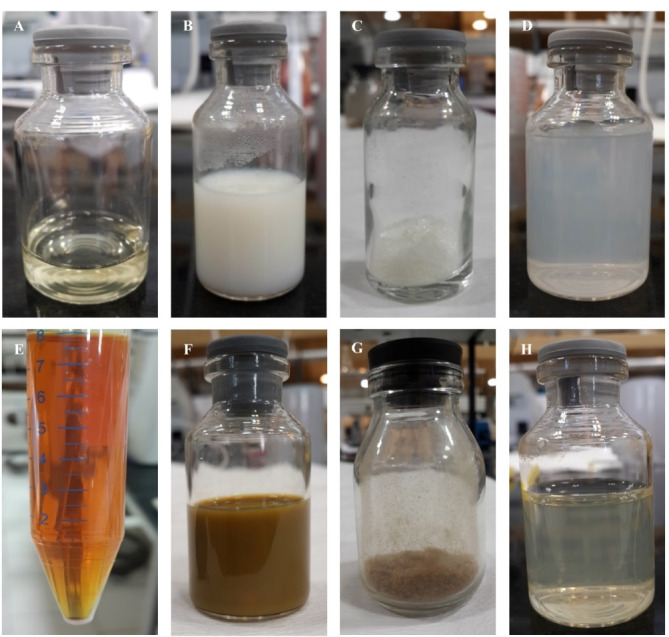
Photographic images taken from different steps of desolvation method considered in this study for the development of BSA and JUG-BSA NPs. An aqueous solution of BSA (250 mg/4 mL) (A), the prepared BSA NPs after the addition of 8 mL ethanol into BSA solution and crosslinking step for 3h with a total volume of 12 mL (B), the lyophilized form of BSA NPs upon purification step (C), the appearance of the redispersed BSA NPs (D), the ethanolic solution of JUG (12.5 mg/8 mL) (E), JUG-BSA NPs prepared by the addition of 8 mL ethanol containing 12.5 mg JUG and after crosslinking process for 3h (F), the lyophilized form of JUG-BSA NPs after purification step (G), and the appearance of redispersed JUG-BSA NPs (H)

**Figure 3:**
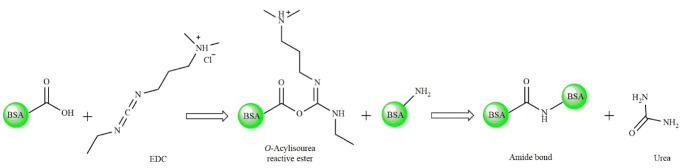
Schematic illustration of the EDC crosslinking reactions

The objective of the current investigation was to obtain BSA NPs for the delivery of JUG. The desolvation method, a simple technique, has been widely used in the encapsulation of various water-soluble or insoluble drugs. In the case of protein-based NPs, they can be fabricated by using multiple proteins such as albumin and gelatin.^36^ Among them, serum albumin is one of the preferred materials for the preparation of protein-based NPs, and to examine the efficiency of serum albumins as an active drug carrier.^37,38^ Accordingly, both BSA and JUG-BSA NPs were prepared using the desolvation technique, in which ethanol was used as a dissolving agent followed by stabilizing them with EDC as a crosslinker agent (see Figure 2 for further details). EDC is a zero space cross-linker forming amide bands between –COOH and –NH_2_ functional groups of amino acids in the prepared NPs. The reaction by-product urea could simply be removed by centrifugation.^31^ Figure 3 shows the photographic images taken from an aqueous solution of BSA, the ethanolic solution of JUG, prepared BSA and JUG-BSA NPs, the lyophilized form of BSA and JUG-BSA NPs, as well as the appearance of redispersed BSA and JUG-BSA NPs. In the preparation process of BSA and JUG-BSA NPs, the amount of free albumin in the supernatants of the separated NPs was determined by Bradford assay and the results showed that the concentration of albumin was not considerable indicating that nearly all of the BSA molecules were incorporated in the prepared NPs. JUG has low solubility in water and for the entrapment of this hydrophobic compound in the albumin NPs, it was dissolved in ethanol and then added to the albumin solution. Others have used the same procedure employed in the present study to prepare PTX-loaded BSA nanoparticles (PTX-BSA NPs).^39^ They dissolved PTX, a water-insoluble anticancer drug, in ethanol and then added to the aqueous solution of albumin to develop PTX-BSA NPs. Finally, the prepared NPs were stabilized by crosslinking reaction using glutaraldehyde as a crosslinker. The obtained drug entrapment and loading efficiencies were 95% and 27%, respectively. In another study, BSA-based NPs of a poorly soluble anticancer drug, 10-hydroxycamptothecin, with encapsulation showed loading efficiency of 90.5% and 57.5%, respectively, using the emulsification technique.^40^ Other technologies such as nab-technology and self-assembly were also successfully employed for the encapsulating of poorly soluble drugs.^36^

###  Particle size and surface charge

**Figure 4:**
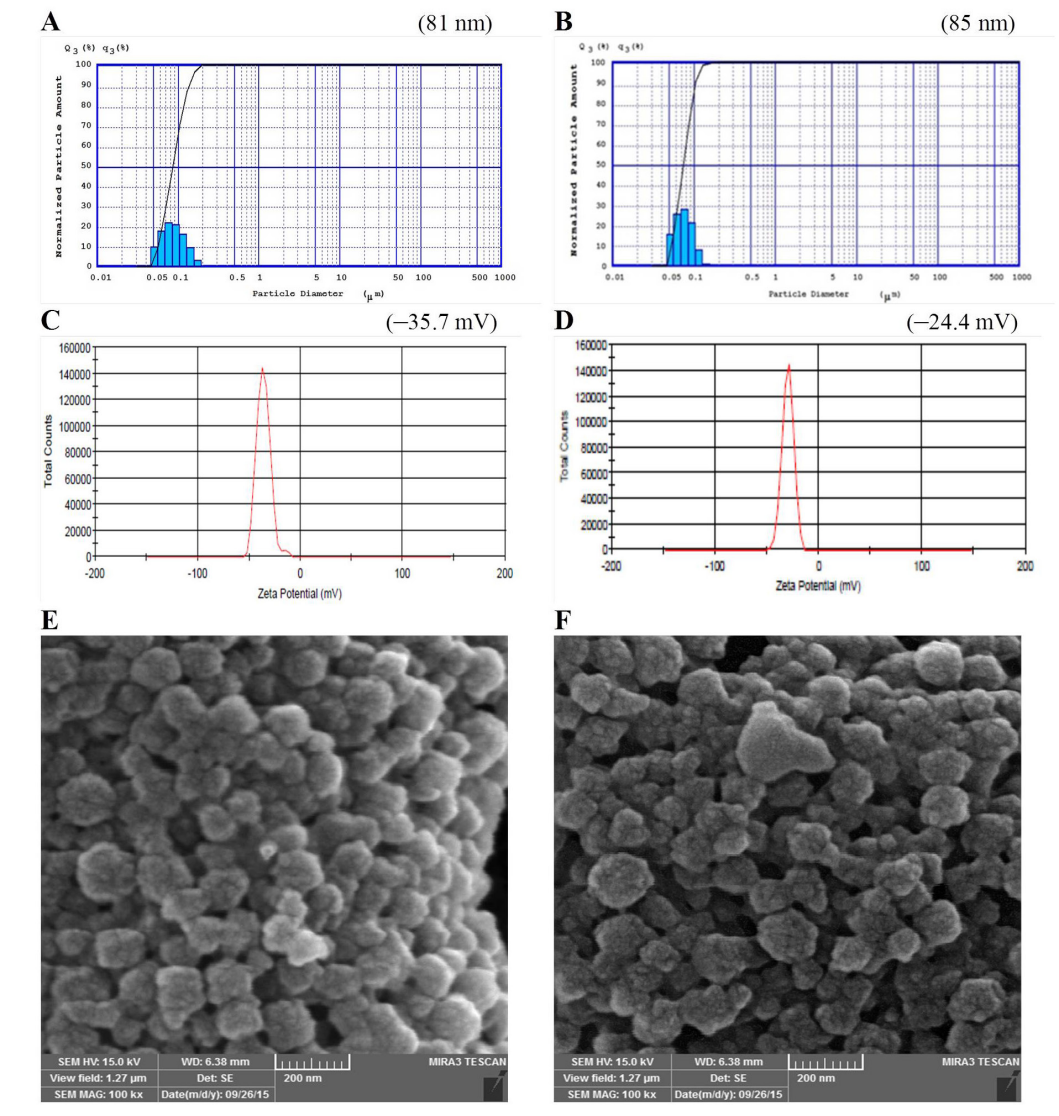
PS (A and B), and ZP (C and D) of BSA NPs and JUG-BSA NPs, respectively. The SEM images of BSA NPs (E) and JUG-BSA NPs (F)

Size and surface charge density significantly influence the distribution of intravenously injected NPs in the tissues and also their clearance by the reticuloendothelial system.^41,42^ Usually, the mononuclear phagocytic system rapidly uptake the NPs with a diameter of more than 200 nm and sequester them in the spleen and liver. The endothelial fenestral formation of the liver presenting caveolae with the size between 50–100 nm filters small NPs below 100 nm. A high proportion of such small particles tend to accumulate in the liver instead of remaining in circulation, regardless of their charge.^43^ A colloidal system with a size between 100–300 nm and narrow size distribution is often used as a drug carrier. However, other features are needed to be taken into consideration; e.g. colloidal stability and surface charge.^44^ In the present study, the PS of the prepared BSA and also JUG-BSA NPs were around 100 nm (81 ± 9 nm and 85 ± 7 nm, respectively) (Figure 4A, B). The polydispersity index was well below 0.2 (0.19 ± 0.05 for BSA NPs and 0.14 ± 0.06 for JUG-BSA NPs), indicating a narrow PZ distribution.

Generally speaking, the net charge is an important parameter because it has influences aggregation, opsonization, and clearance of a drug formulation as well as its stability and circulation time. In a suspension containing NPs with a similar high charge, the inter-colloidal repulsive force between colloids prevents them from the agglomeration and thus, makes it redisperse easily and increases the stability of the formulation.^45^ A minimum value of ± 20 mV for the ZP of NPs is recommended for electrostatic and steric stabilization of the NPs.^46^ BSA and JUG-BSA NPs developed in this study showed a ZP of –35.7 and –24.4 mV, respectively, which is consistent with a stable nanosuspension. Figure 4C and 4D illustrate the ZP of the prepared NPs. The entrapment of JUG in BSA NPs affected the size and ZP of JUG-BSA NPs when compared to that of BSA NPs.

###  Drug loading

To determine the drug loading (DL) capacity, different amounts of JUG were added to the albumin solution during the preparation procedure of JUG-BSA NPs (Table 1). For this purpose, in the first step, 25 mg JUG was weighed (10 fold lower than the amount of albumin) and after dissolving in ethanol and preparing JUG-BSA NPs, the supernatants of the separated NPs were collected and the concentration of unloaded JUG was determined. 12.6 ± 0.6 mg of JUG was loaded in the BSA NPs. In the next steps, the amount of JUG was reduced to 12.5 and 10 mg leading to the loading of 10.3 ± 1.4 mg and 9.4 ± 0.4 mg JUG in the prepared NPs, respectively. These results indicated that approximately 9–12 mg JUG could be loaded onto 250 mg albumin NPs. The EE and loading efficiency of JUG-BSA NPs were also calculated (Table 1). Formulation No. 2 in Table 1 was used in the next experiments because of the compromised balance between DL and EE. The dialysis experiments revealed that JUG NP formulations do not release free JUG into the medium under sink conditions.

###  The morphology of NPs 

The morphology of the prepared BSA and JUG-BSA NPs was investigated using SEM. The SEM images showed that both BSA NPs and JUG-BSA NPs were almost spherical particles in the nanometer range (Figure 4E, F). Additionally, the mean PZ achieved from the SEM measurements was around 100 nm, confirming the results obtained from the laser PZ analyzer.

###  Physical stability

**Figure 5:**
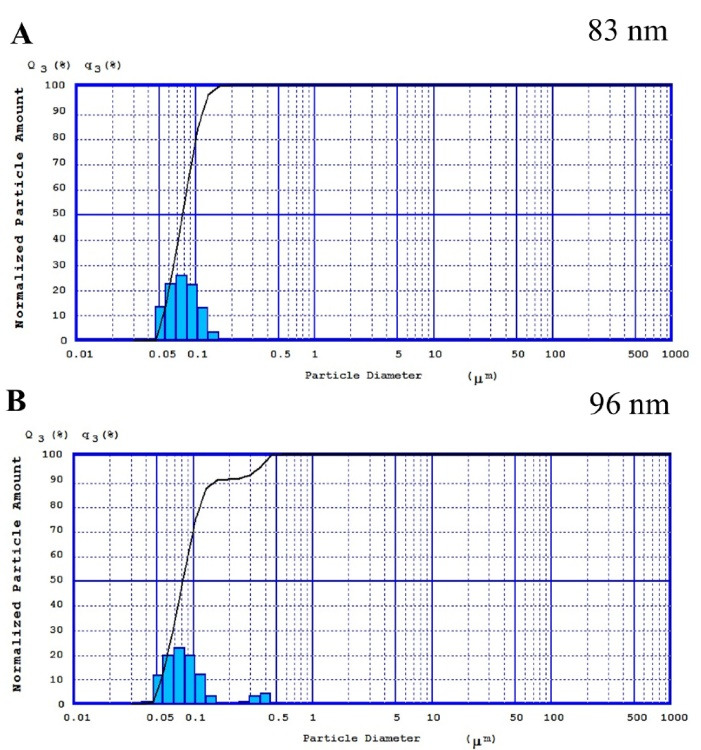
The PZ of the prepared JUG-BSA NPs (A) and the same NPs after 2 weeks of storage at room temperature (B)

Figure 5A shows the PS of JUG-BSA NPs. Figure 5B represents the PS of the same NPs dispersed in PBS and stored at room temperature after 2 weeks. The majority of NPs retained their size (~100 nm) which could be due to the highly uniform surface charge of the particles, and hence, the formulation showed reduced precipitation tendency and enhanced stability. However, the size of a very minor population of NPs changed to around 400 nm during two weeks of storage. This might be resolved by optimizing the storage conditions (using reduced temperature, stabilizers, and lyophilization). The amount of leaked JUG from JUG-BSA NPs was also determined by UV spectroscopy and the results (absorbance below 0.05) showed that after two weeks, the amount of JUG loaded in BSA NPs did not change significantly.

###  Cellular uptake studies

**Figure 6:**
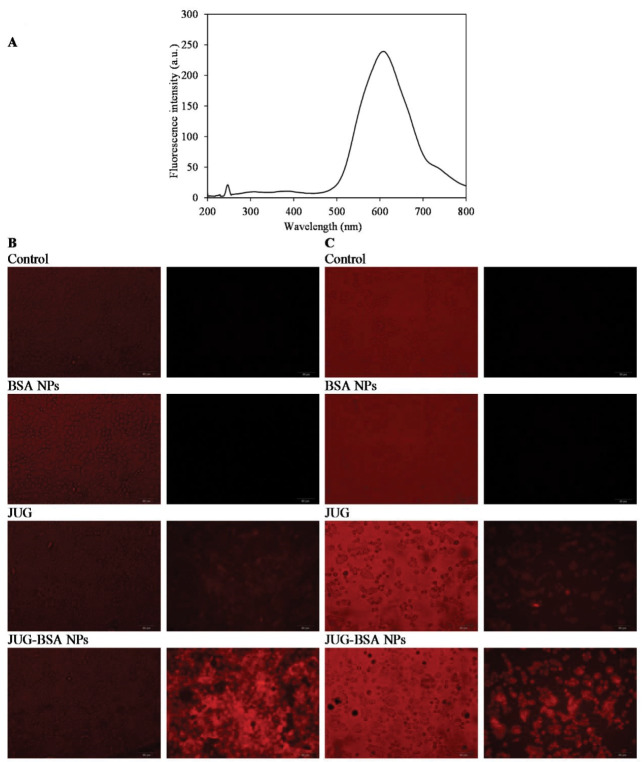
The cellular uptake of JUG, BSA NPs and JUG-BSA NPs by cultured A431 and HT29 cells. The fluorescence spectra of JUG with a concentration of 5 µM excited at 247 nm (A). Monolayers of A431 (B) and HT29 (C) cells were incubated with JUG, BSA NPs and JUG-BSA NPs (1 mg/mL) for 4 h at 37°C and viewed by either phase contrast (left panels) or fluorescence microscopy (right panels)

The cellular uptake of JUG, BSA NPs and JUG-BSA NPs by cultured A431 and HT29 cells was visualized using fluorescence microscopy (Figure 6). To this end, A431 and HT29 cells were incubated with JUG, BSA NPs and JUG-BSA NPs (1 mg/mL) at 37°C for 4 hours. In this study, the fluorescence properties of JUG were used to study the cell internalization without any additional labeling of NPs with fluorescent dyes. Figure 6A shows the fluorescence spectrum of JUG excited at 247 nm. JUG showed a characteristics emission peak around 600 nm. Thus, the fluorescence intensity peak at 600 nm was used to detect JUG. The presence of fluorescence intensity in the cells treated with JUG and JUG-BSA NPs in contrast to the untreated cells or cells treated with BSA NPs used as a control revealed the internalization and uptake of NPs containing JUG by both A431 and HT29 cells. A431 and HT29 cells treated with JUG-BSA NPs exhibited more fluorescence intensity than both A431 and HT29 cells treated with free JUG. As expected, the cells treated with BSA NPs did not show any fluorescence (Figure 6B, C).

###  MTT assay

Among the most broadly distributed quinones, naphthoquinones are described as possessing a wide variety of pharmacological properties, including antifertility, leishmanicidal, antifungal, hypolipidemic, anti-inflammatory, antiatherosclerotic, antibacterial, and antimalarial effects.^47^ Recently, the anticancer activity of naphthoquinones such as plumbagin (5-hydroxy-2-methyl-1, 4-naphthoquinone) and JUG have received considerable attention for their potential use as novel anticancer drugs.

**Figure 7:**
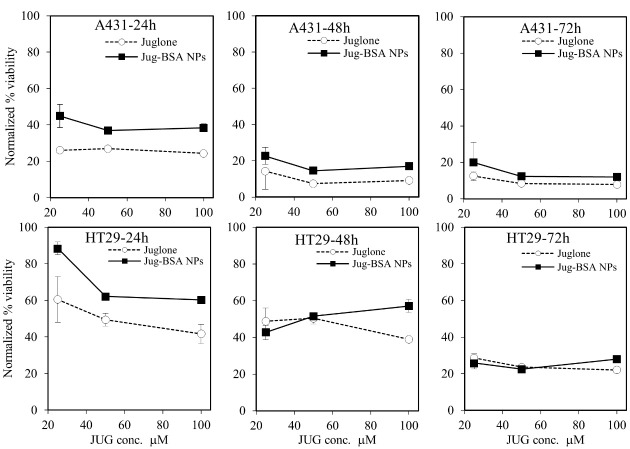
The cytotoxic effects of JUG, BSA NPs and JUG-BSA NPs on A431and HT29 cancer cells. The cells seeded in 96 well plates were treated for 24, 48 and 72 h with different concentrations of JUG, BSA NPs and JUG-BSA NPs in the range of 5–100 µM and the treatments were assayed at least in triplicates

In this study, A431 and HT29 were used to study the cytotoxicity of JUG-BSA NPs. The cultured cells were treated with various equimolar concentrations of JUG preparations over 24, 48, and 72 hours. The viability of cells treated with JUG-BSA NPs (equivalent to JUG concentrations of 25, 50 and 100 µM) was compared with that of free JUG. The cell viability values in the presence of JUG-BSA NPs and JUG were normalized with respect to the cells treated with BSA NPs as shown in Figure 7. BSA NPs had little effect on cell viability, and after 72 hours the viability did not change substantially. Such normalization was performed to make the results obtained for different cells, concentrations, and incubation times comparable. The results indicated that the efficacy of the JUG-BSA NPs depended on the concentration of the loaded JUG, though not very strong, and was also cell-line and exposure-time dependent (Figure 7). JUG-BSA NPs with a concentration of 25 µM had the lowest effect (88.4% viability) on HT29 cells at 24 hours, whereas its effect on A431 cells at the same concentration was significantly higher (44.9% viability) (Table 2). The longer cells were exposed to JUG-BSA NPs, the higher the toxicity. For example, the viability of HT29 cells treated for 24, 48 and 72 hours with JUG-BSA NPs at 25 µM decreased from 88.4 to 42.8 and 25.8%, respectively. The higher concentration of JUG in the NPs formulation conferred increased activity. For instance, JUG-BSA NPs at concentrations equivalent to 50 and 100 µM JUG reduced cell viability of HT29 cells to 60.2 and 60.3% in 24 hours, respectively. More or less, the same trends in terms of incubation time and equivalent JUG concentration were seen for A431 cells (Table 2). In almost all experiments, the toxic effect of JUG-BSA NPs was less than that of JUG. However, this relatively higher safety of JUG-BSA NPs was more pronounced at lower exposure time, i.e., 24 hours incubation time. BSA NPs had little effect on the cell viability, and after 72 hours the viability did not change significantly.

**Table 2:** The results of MTT assay using JUG and JUG-BSA NPs on HT29 and A431 cells. The results are expressed relative to BSA NPs

**Incubation time (h)**	**Cell lines**	**HT29**			**A431**		
	**JUG concentrations (µM)**	**25**	**50**	**100**	**25**	**50**	**100**
	**Formulation**	**Relative cell viability (%)**					
24	Free JUG	60.5	49.4	41.7	26.1	26.9	24.4
	JUG-BSA NPs	88.4	62.2	60.3	44.9	36.9	38.3
48	Free JUG	48.8	50.4	38.9	14.3	7.4	9.1
	JUG-BSA NPs	42.8	51.5	57.1	22.7	14.5	17.0
72	Free JUG	28.5	23.6	22.1	12.5	8.5	7.9
	JUG-BSA NPs	25.8	22.5	28.0	20.0	12.3	12.0

As mentioned, JUG shows low solubility in water and this may limit its application in cancer treatment. Thus, BSA NPs represent promising carriers to overcome this problem. By using BSA NPs, it is anticipated that JUG can be directly delivered to cancerous tissues leading to reduced cytotoxic effects on healthy cells. Hence, JUG-BSA NPs could potentially be used in the treatment of cancer. The cytotoxic effects of JUG obtained in this study on HT29 were in good agreement with the results previously reported in the literature.^17^ In human gastric cancer SGC-7901 cells, the growth inhibition and induction of apoptosis by JUG were attributed to the production of reactive oxygen species, expression of Bcl-2 and Bax proteins, mitochondrial depolarization, the release of cytochrome c into the cytosol and activation of the caspase-3 cascade.^48^

## Conclusion

In summary, JUG-BSA NPs were prepared without using toxic crosslinking agents and emulsifiers, in which JUG was successfully encapsulated in the NPs using the desolvation method. BSA NPs can be used for the delivery of JUG to cancerous cells and may decrease its side effects. JUG-BSA NP formulation may also provide a means for the improvement of water solubility and enhanced delivery of this hydrophobic molecule to the site of action. Furthermore, the results showed that JUG-BSA NPs internalized in A431 more than in HT29 cells and their efficacy depended on the amount of JUG loaded onto BSA NPs and exposure time. The toxic effect of JUG-BSA NPs was less than that of free JUG in almost all experiments. However, this relatively higher safety of JUG-BSA NPs was more noticeable at lower exposure times. Moreover, the prepared JUG-BSA NPs could be useful for targeting particular types of cancer *in vivo* by functionalizing via targeting modifiers, such as ligands for receptors overexpressed in cancer cells.

## Acknowledgments

 This work received funding from the Research Office of Tabriz University of Medical Sciences under the Postgraduate Research Grant scheme for the Ph.D. thesis (No. 92) of AJE. The authors would like to thank Departments of Pharmaceutics and Toxicology at Faculty of Pharmacy at TUOMS for providing particle size analysis and fluorescence microscopy instrumentations. The authors would like to thank Dr. Michael Morris from the University of Sydney for English editing the manuscript.

## Ethical Issues

 Not applicable.

## Conflict of Interest

 The authors declare that there is no conflict of interest.
